# Osteosarcopenia revisited: from mechanisms to management

**DOI:** 10.1093/jbmr/zjag127

**Published:** 2026-08-22

**Authors:** Takayoshi Sasako, Miguel Germán Borda, Marc Sim, Gustavo Duque

**Affiliations:** Department of Medicine, McGill University, Montreal, QC, H4A 3J1, Canada; Lady Davis Institute for Medical Research, Jewish General Hospital, Montreal, QC, H3T 1E2, Canada; Tanaka Diabetes Clinic Omiya, Saitama, 330-0846, Japan; Okachimachi Ohisama Clinic, Tokyo, 110-0016, Japan; Diabetes Research Center, Japan Institute for Health Security, Tokyo, 62-8655, Japan; Department of Diabetes and Metabolic Diseases, Graduate School of Medicine, The University of Tokyo, Tokyo, 113-0033, Japan; Department of Diabetes, Metabolism and Endocrinology, Ichikawa Hospital, International University of Health and Welfare, Chiba, 272-0827, Japan; Centre for Age-Related Medicine (SESAM), Stavanger University Hospital, Stavanger, 4068, Norway; Department of Neurology, Clínica Universidad de Navarra, Pamplona, 31008, Spain; Nutrition and Health Innovation Research Institute, School of Medical and Health Sciences, Edith Cowan University, Perth, WA, 6027, Australia; Lady Davis Institute for Medical Research, Jewish General Hospital, Montreal, QC, H3T 1E2, Canada; Division of Geriatric Medicine, Department of Medicine, McGill University, Montreal, QC, H4A 3J1, Canada; Bone, Muscle and Geroscience Group, Research Institute of the McGill University Health Centre, Montreal, QC, H4A 3J1, Canada

**Keywords:** aging, sarcopenia, osteoporosis, osteokines, myokines, hormone, nutrition, exercise

## Abstract

Sarcopenia and osteoporosis are characterized by insufficient mass and function of skeletal muscle and bone, and often co-occur in older persons, a condition called osteosarcopenia. Loss of bone and skeletal muscle mass and function is driven by multiple risk factors that activate the hallmarks of aging. Recent genome-wide association studies in humans and animal models suggest pivotal roles for mitochondria, the nucleus, and nutrient anabolism, which are closely associated with the hallmarks of aging, as well as for osteokines and myokines, in the development of osteosarcopenia. Osteosarcopenia is associated with a higher risk of adverse outcomes, such as falls, fractures, depression, and frailty, and consequently with mortality. Diagnosis of osteosarcopenia involves various imaging techniques to assess BMD and skeletal muscle mass, as well as the assessments of physical performance or muscle function. Moreover, a comprehensive evaluation of medical history, medications, and laboratory data is important for identifying potential contributing factors and assessing risk. To manage and treat osteosarcopenia, a multimodal approach, such as exercise therapy, nutritional supplementation, and pharmacotherapies, is required. Future prospects include technological innovations such as artificial intelligence and the identification of novel biomarkers based on multi-omics analyses, which will help to enhance early detection of this condition. Emerging drugs that positively regulate bone and muscle mass are under development and are expected to contribute to individually optimized care for osteosarcopenia.

## Introduction

Skeletal muscle attaches to bone and interacts closely with it both mechanically and by secreting hormones and metabolites. Such regulation is essential for normal development and the maintenance of homeostasis,^1^ and it is therefore highly likely that aging in one tissue influences that in another. Indeed, sarcopenia and osteoporosis are age-related conditions of muscle and bone that co-occur in humans.^2^^,^^3^ Both osteoporosis and sarcopenia are linked not only to various age-related diseases, including cardiometabolic conditions and frailty (defined as a systemic vulnerability to external stressors),^4^ but also to mortality. Furthermore, they place significant burdens on health expenditure, which is expected to increase as the incidence of these diseases rises in aging societies.^5^^,^^6^

Aging causes dysfunction in various tissues through changes in the hallmarks of aging, defined as “fundamental biological processes that accumulate over time and lead to a progressive decline in physiological function.”^7^ Alterations of these hallmarks in muscle and bone are associated with a loss of their mass and function (Figure 1).^7–9^ Osteoporosis and osteopenia are characterized by reduced BMD, sarcopenia by a decline in skeletal muscle mass, strength, or physical performance, and osteosarcopenia is a clinical syndrome characterized by the simultaneous presence of these conditions.^6^

**Figure 1:**
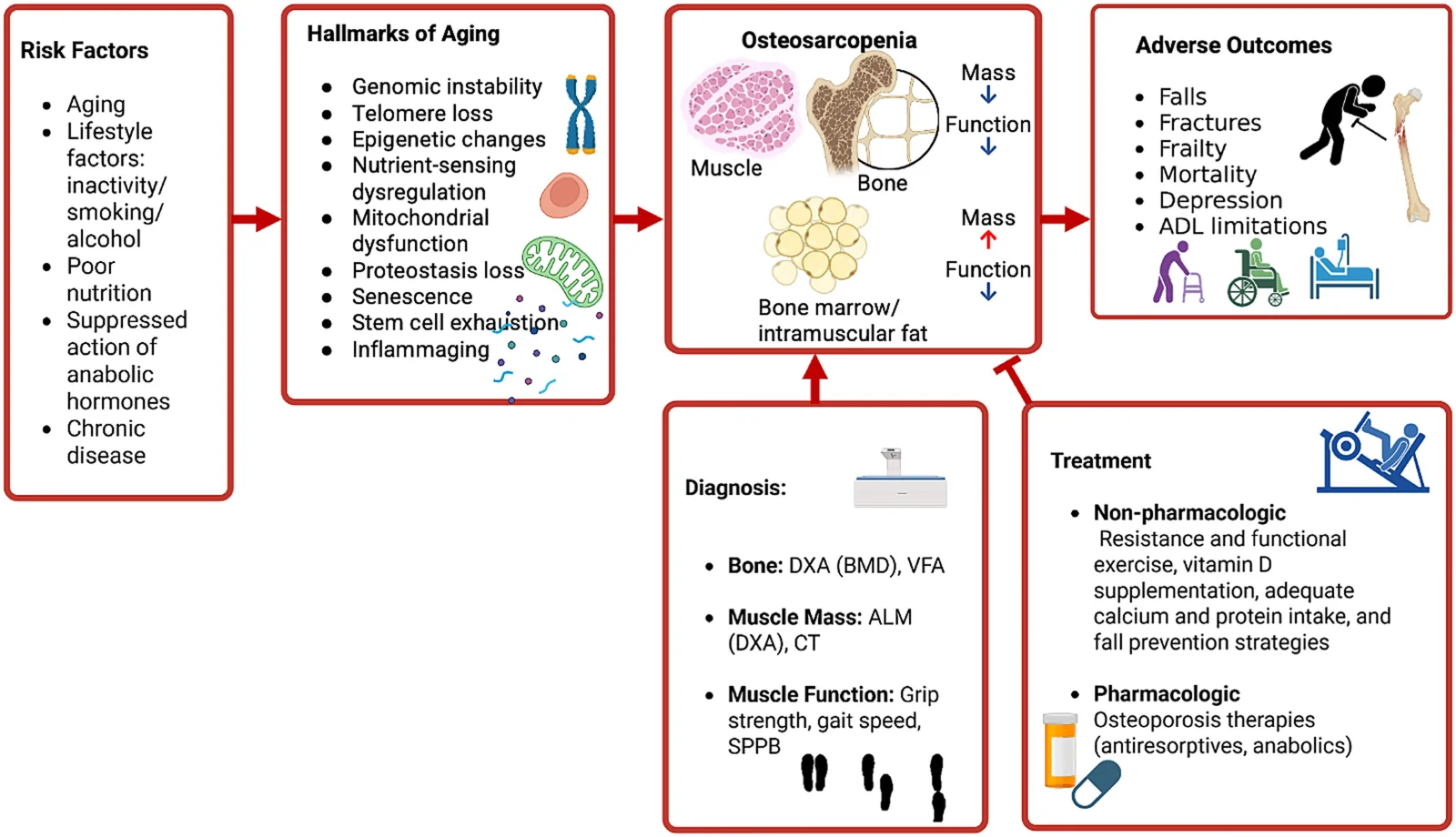
Osteosarcopenia from bench to bedside. In the presence of predisposing risk factors, alterations in hormones, adipokines, and bone–muscle crosstalk disrupt the biology and function of bone and muscle cells, promoting a catabolic state accompanied by impaired anabolic processes. These biological changes lead to the development of osteopenia/osteoporosis and sarcopenia, which, when present concurrently, constitute osteosarcopenia. The diagnosis of osteosarcopenia integrates clinical assessment with imaging and functional evaluation. Therapeutic interventions, including both non-pharmacological and pharmacological approaches, aim to restore the balance between anabolic and catabolic pathways, thereby improving bone and muscle health and reducing the risk of adverse clinical outcomes (Created with BioRender).

Tissue mass is regulated by cytokines and hormones that modulate nutrient metabolism. These regulatory mechanisms tend to become more catabolic with age, even in the presence of anabolic stimulation, particularly in bone and skeletal muscle. For example, in older individuals, bone growth in response to mechanical loading and anabolic hormones such as insulin-like growth factor (IGF)-1 and growth hormone is impaired.^10^ In skeletal muscle, protein synthesis in response to nutrient overload, especially amino acids, is reduced, although baseline synthesis remains unchanged,^11^ and protein synthesis in response to insulin, another anabolic hormone, might be impaired.^12^ Moreover, aging is associated with lower circulating anabolic hormone levels, decreased mechanical loading from inactivity, and reduced nutrient supply from undernutrition (Figure 1). The combination of insufficient anabolic stimulation and resistance to anabolic stimulation leads to enhanced catabolism in these tissues, which is one of the major features of osteosarcopenia.

By contrast, aging is associated with ectopic fat accumulation, including intramuscular and bone marrow fat infiltration, and loss of function of adipose tissue (Figure 1).^9^^,^^13^^,^^14^ Interestingly, such anabolic changes in adipose tissue are involved in the development of catabolic osteosarcopenia via lipotoxicity. Free fatty acids released from adipose tissue into the blood are a major substrate for oxidative phosphorylation in skeletal muscle mitochondria, especially in slow-twitch muscles that are rich in mitochondria and well suited to endurance exercise. However, prolonged exposure to excessive free fatty acids may contribute to lipotoxicity, which could hasten skeletal muscle loss.^14^ Additionally, glycolysis, which involves glucose catabolism, generally plays a crucial role in ATP production in osteoblasts and osteoclasts. The role of free fatty acids as an energy source in bone remains generally unclear,^15^ and elevated free fatty acid levels could accelerate bone loss through lipotoxicity.^16^

Despite increasing recognition of osteosarcopenia, its conceptual framework remains incomplete. Previous reviews have largely focused on its epidemiology and clinical consequences or have examined bone and muscle loss in parallel, with limited integration of the biological mechanisms linking these tissues. In particular, the balance between catabolism and anabolism varies widely across tissues but has not been consistently synthesized. Moreover, recent insights from genetic studies in humans and genetically modified experimental models have yet to be fully translated into clinically relevant frameworks. As a result, osteosarcopenia, despite its clinical and societal consequences, remains inconsistently defined and underrecognized as a unified condition in clinical practice.

In this review, we aim to provide an integrated up-to-date perspective on osteosarcopenia as a systemic, age-related syndrome. We first outline the definition, epidemiology, and clinical relevance, and then synthesize current evidence on the biological mechanisms linking bone and muscle, with particular emphasis on the balance between anabolism and catabolism and inter-tissue crosstalk. Finally, we discuss diagnostic approaches and management strategies, highlighting how emerging mechanistic insights may inform more targeted and individualized clinical care.

## Clinical significance of osteosarcopenia

Although often studied separately, osteoporosis and sarcopenia commonly co-occur in aging populations,^17^ aligning with the age-related changes in bone and muscle described above and increasing their clinical significance in older adults. A study involving 17 891 adults from various ethnicities in the USA and China found that the sarcopenia phenotype, characterized by reduced muscle strength and low muscle mass, was linked to a higher risk of osteopenia or osteoporosis (OR: 1.87, 95% CI: 1.09-3.20).^18^ A 2023 meta-analysis of 66 studies (*n* = 64 404, with a mean age ranging from 46.6 to 93 yr) showed that nearly 1 in 5 individuals had osteosarcopenia (18.5%, 95% CI: 16.7-20.3, I2 = 98.7%).^19^ Notably, the prevalence was higher among women (19.4%, 95% CI: 16.9-21.9, I2 = 98.5%) compared to men (15.3%, 95% CI: 13.2-17.4, I2 = 97.6%). Many of these studies included Asian participants, who exhibited nearly twice the prevalence of osteosarcopenia compared to Europeans (21.6% vs 10.9%). This aligns with other research reporting a higher prevalence of sarcopenia among Asians compared to Whites,^18^ though differences in BMD are less clear.^20^ The meta-analysis also noted that most studies were cross-sectional (*n* = 48, 36 933 participants), followed by prospective (*n* = 11, 25 319 participants) and retrospective (*n* = 6, 1117 participants) cohort studies, as well as a single case–control study.

Importantly, the coexistence of bone and muscle loss confers a compounded risk profile and portends serious clinical consequences. Since osteoporosis and sarcopenia have both been individually associated with a range of adverse clinical outcomes, a combination of these conditions, often called the “hazardous duet,” is likely to substantially increase the risk of these adverse outcomes and worse prognosis^21–41^ (eg, fractures,^24^^,^^25^^,^^27–31^ frailty,^32–35^ depression,^36^^,^^37^ activities of daily living limitations,^38–40^ and mortality^23^^,^^38–41^) (Figure 1). The meta-analysis of the 48 studies mentioned above found that individuals with osteosarcopenia had a higher risk of falls (HR = 1.54, 95% CI: 1.20-1.97; I2 = 1.0%, 3 studies), fractures (HR = 2.13, 95% CI: 1.61-2.81; I2 = 67.8%, 7 studies), and mortality (HR = 1.75, 95% CI: 1.34-2.28; I2 = 0.0%, 5 studies) compared to those without osteosarcopenia.^20^ Another recent meta-analysis showed that older adults with osteosarcopenia have roughly twice the risk of fractures and a 75% increased risk of all-cause mortality compared to peers with normal bone and muscle mass.^19^ Moreover, individuals living with osteosarcopenia tend to experience faster functional decline, often resulting in loss of independence and a greater need for institutional care; a 5-yr cohort study in Japan reported a 33% incidence of new disability among osteosarcopenic individuals vs 6% in those with neither condition.^42^

Navigating the literature on osteosarcopenia is complicated by the numerous ways it has been defined. In brief, some studies include individuals with osteopenia and/or osteoporosis (based solely on BMD T-score, without fracture history), along with various definitions of sarcopenia that consider different cut-points for appendicular lean mass (ALM) and muscle strength (eg, hand grip strength, 5× chair stand test). More recently, some studies have not considered muscle mass and instead use the term probable osteosarcopenia.^39^^,^^40^ This is likely due to the Sarcopenia Definitions and Outcomes Consortium,^43^ which suggests that weak muscle strength may define probable sarcopenia. It is also common for studies to compare osteosarcopenic individuals to those without either condition, presenting as their referent group.^40^^,^^44^ This makes it much harder to disentangle the additive effect of osteosarcopenia vs either disease alone on clinical outcomes. This issue was examined in a study of 1575 community-dwelling older men (≥70 yr). The prevalence of osteosarcopenia was 8%, while 34% had osteopenia/osteoporosis alone and 7% had sarcopenia alone. The study found increased risks for falls (IRR: 1.41, 95% CI: 1.02-1.95) and fractures (HR: 1.87, 95% CI: 1.07-3.26) in men with osteosarcopenia compared to those without either condition. However, no such association was observed when comparing men with osteosarcopenia to those with only one condition.^45^ These findings contrast with another research from the Osteoporotic Fractures in Men study (*n* = 5544, mean age = 73.7 yr),^46^ which indicated that men with both low BMD and sarcopenia had a higher risk ranging from 1.1 to 1.7 times, compared to men without any condition or with only one. Furthermore, a recent meta-analysis reported mixed findings in the context of falls, using both the relative excess risk due to interaction and the multiplicative interaction ratio to assess whether osteosarcopenia confers a greater risk of incident falls and fractures than low BMD or sarcopenia alone. However, these findings should be interpreted with caution, as the analysis included only 7 studies overall and just 3 studies that evaluated falls, limiting the statistical power to detect interaction effects and highlighting the need for additional well-powered studies.^47^

Alternatively, because older women are reported to have a higher risk of osteopenia/osteoporosis and sarcopenia compared to their male counterparts, women are a particularly vulnerable population to osteosarcopenia. It has been reported that 50%-58% of peri- or postmenopausal women with osteoporosis also have sarcopenia.^48^ Such findings are likely exacerbated by declining estrogen levels, which negatively affect both bone and muscle.^49^ These hypotheses are supported by a study reporting that 58% of postmenopausal women who had a hip fracture in the last month also presented with sarcopenia.^50^ In the Study of Osteoporotic Fractures in Women (*n* = 1114, mean age 78 yr), women with low BMD and sarcopenia (HR: 2.27, 95% CI: 1.37-3.76), as well as women with low BMD alone (HR: 2.62, 95% CI: 1.74-3.95), had a greater risk of fracture compared to women with normal BMD and no sarcopenia. Surprisingly, women presenting only with sarcopenia did not show an increased fracture risk.^46^ Despite some limitations raised above, it is clear that the combination of weakened muscle and bone strength is highly detrimental in the context of falls and fractures.

## Clinical assessment of muscle and bone health

Diagnosis of osteosarcopenia usually involves a combination of imaging techniques and physical assessments (Table 1).^6^^,^^51^^,^^52^ Furthermore, a thorough evaluation of medical history and risk assessment is expected to be highly useful (Figure 1).

**Table 1:** How to assess osteosarcopenia in clinical practice.

		**Modalities and details**
**Diagnosis**	BMD^a^	DXA
	Muscle (lean) mass	DXA and BIA
	Physical performance	Handgrip strength testing, five-time chair stand test, gait speed assessment, and SPPB
**Other investigations**	Screening of sarcopenia	SARC-F
	Past history	(Low-energy) fracture^a^, rheumatoid arthritis^a^, secondary osteoporosis^a^, and diabetes mellitus
	Medications	Corticosteroid^a^ and other potentially catabolic drugs
	Family history	Hip fracture^a^
	Life history and others	Smoking^a^, alcohol intake^a^, weight history, and nutritional history
	Laboratory data	25OHD, calcium, phosphate, renal/liver function, TSH, PTH, albumin, total protein, prealbumin, lymphocyte count, plasma glucose, and glycated hemoglobin

a Risk factors for FRAX score calculation (BMD is optional).

Abbreviations: BIA, bioelectrical impedance analysis; SPPB, short physical performance battery.

### Bone density and osteoporosis assessment

DXA remains the gold standard to assess BMD. DXA is typically measured at the LS and proximal femur. A T-score of ≤−2.5 at these sites confirms a diagnosis of osteoporosis, while values between −1.0 and −2.5 fall within the osteopenic range.

### Muscle mass assessment

DXA is the most commonly used method to quantify ALM and derive indices like ALM/height,^2^ which is called skeletal muscle mass index (SMI).^53^ Bioelectrical impedance analysis (BIA) is an alternative for estimating muscle mass when DXA is not available; modern multifrequency BIA devices show reasonably good agreement with DXA in estimating skeletal muscle mass.^51^ Anthropometric surrogates (such as calf circumference) can be used for initial screening in resource-limited settings, but precise measurement is preferred.^52^ It should be noted that the diagnosis of sarcopenia is not based on muscle mass alone, low muscle strength must also be present.

### Physical performance assessment

Methods based on the EWGSOP2 for detecting low muscle function and performance include handgrip strength testing, the five-time chair stand test, gait speed assessment, and the Short Physical Performance Battery (SPPB). Handgrip strength is considered the primary measure of muscle strength, while gait speed and chair rise performance are key indicators of functional capacity. A gait speed of <0.8 m/s, a chair stand time of ≥15 s, or an SPPB score of ≤8 suggest impaired physical performance and are linked to an increased risk of adverse outcomes, such as falls and mobility loss.^51^

### History taking and laboratory investigations

Clinical assessment of osteosarcopenia should begin with a detailed medical history, with particular attention to previous low-trauma fractures. The FRAX score, which reflects skeletal fragility that may predispose to fall-related fractures associated with impaired muscle function, may justify clinical management. FRAX is simple to calculate and generally does not require laboratory investigations; BMD is not mandatory in all cases. Because vertebral fractures are frequently asymptomatic, lateral spine imaging or DXA-based vertebral fracture assessment (VFA) should be considered to identify unrecognized fractures that may influence treatment decisions and prognosis.^54^ For sarcopenia, the SARC-F is a questionnaire to screen for sarcopenia by testing indicators of muscle condition, such as strength, assistance with walking, rise from a chair, climb stairs, and falls.^55^ Although SARC-F captures predominantly functional characteristics, other aspects including weight history and lifestyle factors, such as smoking and drinking, should be considered, as they play an important role for muscle and bone health.

Medications that could be associated with increased musculoskeletal catabolism are also important. For example, corticosteroids—one of the factors used in calculating the FRAX score—are well known to promote catabolism in both bone and skeletal muscle, making osteoporosis and myopathy major adverse effects of corticosteroid therapy.^56^ An elevated urea nitrogen (UN)/creatinine ratio may serve as a biomarker of amino acid catabolism associated with steroid myopathy.^57^ Sodium-glucose co-transporter 2 (SGLT2) inhibitors are also known to promote catabolism and may also be associated with bone and muscle loss, although this remains controversial.^58^ The effects of incretin-based drugs on bone and muscle are also controversial because they are associated with weight reduction but may also stimulate anabolism in these tissues.^59^ More patients will likely be on corticosteroids for longer periods, not only for autoimmune diseases but also as part of chemotherapy for malignancies or immunosuppressive therapy following organ transplants. Similarly, SGLT2 inhibitors and incretin-based drugs are used not only for diabetes but also for heart failure prevention and kidney protection. Consequently, it is increasingly important to pay close attention to the risk of secondary osteosarcopenia related to these medications.^57^^,^^60^

Although no laboratory tests are specific for osteosarcopenia, routine blood work can help identify secondary causes and contributing factors. Assessment usually includes 25OHD, calcium, and phosphate.^6^ Renal and liver function should be evaluated to exclude metabolic or systemic contributors to bone and muscle loss.^6^ Screening for secondary osteoporosis involves checking PTH and thyroid-stimulating hormone (TSH) levels to rule out parathyroid or thyroid disorders.^6^ High levels of PTH and TSH have been associated with osteosarcopenia in older persons.^61^^,^^62^ Low serum albumin or total protein levels may indicate malnutrition, affecting both bone and muscle.^6^ Prealbumin and lymphocyte count are also expected to reflect nutritional status. Plasma glucose and glycated hemoglobin tests help to screen for diabetes, in which insulin-mediated nutrient anabolism is impaired.

Finally, there is increasing interest in inflammatory mediators, such as high-sensitivity C-reactive protein (hs-CRP) and interleukin-6 (IL-6), which are often elevated in older adults with muscle wasting and bone loss. These markers, along with emerging candidates such as IGF-1, a key anabolic hormone, RANKL, an osteokine to enhance bone resorption or bone catabolism, and myostatin, a myokine of potential importance, may eventually assist in risk stratification or monitoring response to interventions, although further validation is needed before they can be used routinely in clinical practice.^21^^,^^63^

## Imaging modalities in osteosarcopenia

Accurate measurements of bone density and muscle morphology are crucial for both diagnosis and monitoring of osteosarcopenia (Figure 1). A variety of imaging techniques are employed, each with strengths and limitations.

### DXA

DXA is considered a cornerstone imaging modality for assessing osteosarcopenia.^64^ DXA is widely available, quick to perform, and exposes patients to minimal radiation. Beyond standard BMD and body composition, it also offers additional insights such as the trabecular bone score (TBS), a texture-based index derived from DXA images that serves as a surrogate of trabecular bone microarchitecture and improves fracture risk prediction when used alongside BMD. Advanced software can further analyze bone geometry (eg, hip structural analysis) and estimate limb muscle volume, though these are mainly research tools. However, DXA has limitations; it measures muscle quantity but not quality, and cannot detect fat infiltration within muscle or bone, which is a key finding in osteosarcopenia.^65^ Its accuracy can also be influenced by hydration status or conditions such as edema, which may lead to an overestimation of lean mass.

### Computed tomography

Computed tomography scans provide high-resolution measurements of both bone and muscle and have become valuable in osteosarcopenia research. Quantitative CT (QCT) can measure volumetric BMD of the spine or hip with great accuracy, and peripheral QCT (pQCT) or HR-pQCT can detail bone microarchitecture (cortical thickness, trabecular density) in the radius or tibia.^66^ For muscle, axial CT images—typically at the lumbar L3 level—are used to quantify muscle cross-sectional area and quality. The cross-sectional area of the psoas or the total abdominal musculature at L3 strongly correlates with whole-body muscle mass and is a recognized surrogate for sarcopenia in numerous studies.^53^ Importantly, CT can assess muscle quality by measuring radiologic attenuation of muscle tissue in Hounsfield units (HU). Lower muscle density (eg, due to fat infiltration) manifests as lower HU values and is associated with weakness and worse clinical outcomes.^53^ One strategic advantage of CT is opportunistic screening: many older adults undergo CT imaging for unrelated reasons (abdominal CT for cancer staging, chest CT, and so on), and those scans can be retrospectively analyzed to evaluate muscle mass and bone status without extra radiation.^67^ For example, an abdominal CT done for colon cancer follow-up can yield the patient’s L3 muscle area and density (sarcopenia assessment) and also lumbar vertebral trabecular density (osteoporosis assessment). This opportunistic use of existing CT scans is an emerging approach to identify osteosarcopenia in patients who might otherwise not be screened.^68^ The downside of CT is radiation exposure and cost, which preclude its use solely for screening in most cases. However, when available, CT imaging provides a very detailed picture: it can confirm osteoporosis (by low vertebral HU or detecting occult fractures) and sarcopenia (by reduced muscle cross-sectional area and density), thereby richly characterizing the osteosarcopenic phenotype.

### Magnetic resonance imaging

Magnetic resonance imaging is considered a gold-standard for muscle volume and quality assessment due to its excellent soft tissue contrast and lack of radiation. Whole-body or regional MRI can directly measure muscle volume and cross-sectional area with high precision, and can quantify intramuscular fat infiltration using techniques like Dixon imaging.^69^ For bone, MRI (particularly high-resolution MRI of the peripheral skeleton) can evaluate trabecular architecture and even predict biomechanical strength.^70^ However, in clinical practice, MRI is not routinely used for diagnosing osteoporosis or sarcopenia because it is expensive and time-consuming.

### Ultrasound

Ultrasound can measure muscle thickness, cross-sectional area, and echo intensity (a proxy for muscle quality) in various muscle groups, commonly the quadriceps or calf. It is portable, inexpensive, and radiation-free, making it attractive for bedside or community screening of muscle health.^71^ Muscle thickness on ultrasound correlates well with DXA-derived lean mass and can discriminate sarcopenia; for example, a certain cut-off in rectus femoris thickness may predict low ALM.^72^ The main limitations of ultrasound are operator dependence and lack of universal standardization. Moreover, QUS has long been used to assess bone properties, particularly at peripheral sites such as the calcaneus. Emerging techniques, such as ultrasound CT and their potential future role are promising, particularly in combination with artificial intelligence (AI)-based image analysis.

## Genetic determinants of osteosarcopenia in humans

The genetic determinants of a disease are expected to provide insight into the mechanisms that underlie its development. To this end, genome-wide association studies (GWASs) have been conducted extensively over the past decades, revealing the genetic determinants of osteoporosis and sarcopenia, although there is a possibility that even a gene or pathway causal for a disease might be overlooked by GWASs.^73^ Here, we focus on single nucleotide polymorphisms (SNPs) whose effect allele has been shown, by previous GWASs, to be associated with both BMD and sarcopenia-associated traits with the same direction. These SNPs are expected to help elucidate molecular basis that osteoporosis and sarcopenia have in common.

Genome-wide association studies of BMD have been reported since 2007,^74^^,^^75^ whereas GWASs of lean mass have been reported since 2009,^76^ both of which have identified an enormous number of SNPs associated with these traits. Genome-wide association studies of grip strength^77^ and GWASs of gait measurement traits^78^ have also been reported in the past decade. The overlap between SNPs associated with BMD and SNPs associated with sarcopenic traits has not been fully elucidated, but there are at least two SNPs whose effect allele is associated both with BMD and lean mass in the same direction in individuals of European ancestry: rs9972653 located in an intron of the *FTO* gene and rs9379084 located in an exon of the *RREB1* gene.

rs9972653(G) in the *FTO* gene is associated with both decreased heel BMD^79^ and decreased lean body mass.^80^ Interestingly, the allele is linked to a higher risk of metabolic syndrome in East Asians,^81^ although it is also associated with decreased fat mass^80^ and a higher self-reported walking pace^82^ in Europeans. Moreover, several other SNPs in or near the *FTO* gene are linked to osteosarcopenic traits: rs1421085 with lean mass and hand grip strength^83^^,^^84^; rs9924983 and rs8063057 with heel BMD^85^; rs113191842 and rs9930333 with lean body mass^80^^,^^83^; rs11642015 with hand grip strength^84^; and rs8061064 with low hand grip strength.^86^ The *FTO* gene was identified as an obesity-related gene in the late 2000s.^87^ The FTO protein is expressed in tissues ubiquitously, mainly localized in the nucleus, and is believed to be involved in DNA demethylation.^88^^,^^89^ Furthermore, a human study suggests that FTO may be involved in regulating mitochondrial thermogenesis and lipid storage.^90^ These findings highlight the potential importance of FTO and its interaction with fat, bone, and skeletal muscle in the development of osteosarcopenia.

rs9379084(A) is a missense allele in the *RREB1* gene. The allele is known to be associated with decreased heel BMD,^79^^,^^85^ and has recently been reported to be linked to decreased lean body mass.^83^ It is also associated with lower body height^80^ and higher BMI^91^ in Europeans. RREB1 protein is also located in the nucleus, serving as a corepressor involved in chromatin remodeling.^92^ Specifically, RREB1 is known as a repressor of the androgen receptor (AR) gene,^93^ which encodes a nuclear receptor that mediates anabolic androgen signaling which is negatively associated with osteosarcopenic traits in general,^94^ and as an activator of insulin genes.^95^ Indeed, rs9379084(A) is also associated with lower sex hormone-binding globulin levels, higher bioavailable testosterone levels,^96^ and a reduced risk of type 2 diabetes.^97^ These findings suggest that the locus may be involved in regulating anabolic signaling, although the mechanisms influencing various traits may be complex and are yet to be fully understood. Notably, the allele is associated with heel BMD and BMI in both women and men.^79^^,^^91^

## Implications from genetic model mice of osteosarcopenia

To elucidate the precise mechanisms underlying the development of osteosarcopenia, various animal models have been developed. Here, we focus on genetically modified mouse models that exhibit both osteoporotic and sarcopenic phenotypes, because they serve as strong evidence to link the genes of interest with molecular mechanisms of osteosarcopenia. Systemic KO mouse models, similar to GWASs in humans, provide insight into mechanisms shared by osteoporosis and sarcopenia, while tissue-specific KO mouse models offer clues about muscle–bone interactions.

Regarding the two genes encoding a nuclear protein mentioned above, the systemic *Fto* KO mice display bone and skeletal muscle loss, as well as growth retardation,^98^ inconsistent with GWAS results in humans. Homozygous *Rreb1* KO mice are embryonic lethal, while heterozygous *Rreb1* KO mice serve as a model of Noonan syndrome rather than osteosarcopenia.^99^ Similarly, systemic KO mice of the *Lmna* gene, which encodes lamin A/C proteins that form the nuclear membrane and are associated with progeria and other diseases in humans,^100^ exhibit severe myopathy, bone loss, and ectopic fat infiltration in bone and skeletal muscle, but they also exhibit growth retardation and do not survive beyond 8 wk.^101–103^

Nevertheless, systemic ablation of other nuclear proteins, ZMPSTE24, a metalloproteinase responsible for processing and maturing lamin A protein,^104–106^ or ERCC1, an endonuclease involved in DNA repair,^107–109^ results in osteosarcopenic phenotypes at ages 2-5 mo and death in half a year. Notably, the phenotypes of the *Ercc1*^Δ/−^ hypomorphic mice are partially reversed by ablation of NF-kB, the key transcription factor regulating inflammation, and by administration of soluble activin receptor type IIB (ActRIIB) to block myostatin/activin signaling, respectively.^108^^,^^109^

Mitochondrial dysfunction is believed to accelerate aging.^7^ A mouse model with a large-scale deletion in the mtDNA, along with a model carrying a mutated *Polg*, a mitochondrial DNA polymerase, exhibits phenotypes such as muscle atrophy and bone loss around 10 mo of age and typically die by about 1 yr.^110^^,^^111^ Systemic deletion of *Fam210a*, which encodes a protein mainly expressed in the mitochondria of skeletal muscle and other tissues but not in bone, also results in muscle loss and bone loss by 2 mo of age.^112^

Anabolic hormones include insulin, IGF-1, and androgens, and in contrast with systemic *Ir* or *Igf1r* KO mice,^113^^,^^114^ systemic *Ar* KO mice are not neonatal lethal and remain viable. Osteopenia and muscle loss are observed in the male *Ar* KO mice of multiple strains,^115–118^ which are not necessarily replicated in others.^119^

IGF-1 is secreted locally from various tissues, whereas sclerostin (SOST) is an osteokine derived from bone. It negatively regulates bone mass, and systemic *Sost* KO mice exhibit increased bone mass between 8 and 30 wk of age. They also display increased lean mass, although of marginal significance.^120^^,^^121^ FGF23 is an osteokine that negatively regulates phosphate metabolism. Systemic *Fgf23* KO mice exhibit ectopic calcification and growth retardation and cannot survive beyond 3 mo. They also show skeletal muscle wasting and bone loss, which are considered to be dependent on Klotho, composing the signaling receptor of FGF23, and vitamin D.^122–124^ Myostatin (MSTN) is known as a myokine that negatively regulates muscle mass and possibly bone mass. Systemic *Mstn* KO mice show not only increased skeletal muscle mass but also increased bone mass, although the bone phenotypes are atypical in some aspects.^125–128^

Conditional KO mice also serve as models of osteosarcopenia. The skeletal muscle-specific inducible *Fam210a* KO mice show similar muscle phenotypes to those of the systemic *Fam210a* KO mice mentioned above. The osteopenic phenotypes are observed only slightly, mainly in males, but increased muscle MMP12 expression is proposed as a link between muscle and bone.^112^ Skeletal muscle-specific KO of *Gadd45gip1*, which encodes a nuclear protein that regulates mitochondrial translation called Crif1, leads to muscle and bone loss. These phenotypes are partly reversed by antagonizing the CXCL12-CXCR4 axis to reduce bone marrow inflammation, suggesting an interaction between skeletal muscle, bone marrow, and bone.^129^

Skeletal muscle-specific *Ar* KO mice exhibit bone loss at 3 mo of age, followed by muscle loss at 1 yr. These phenotypes are observed only in males, and suppressed polyamine biosynthesis in skeletal muscle is proposed as a mechanism underlying the bone loss.^130^ Moreover, male mice with *Ar* knocked out in a manner specific to satellite cells, or progenitor cells of myocytes, were reported to show a reduction in perineal muscle mass at the age of 12 wk.^131^ Interestingly, inducible satellite cell-specific *Ar* KO mice showed bone loss 7 wk after the deletion.^132^

Lastly, skeletal muscle-specific deletion of *Akt1*/*2*, the key downstream kinases of insulin/IGF-1 signaling, leads to muscle and bone loss that appears before 2 mo of age, and a shortened lifespan by about 10%, with increased deaths from debilitation. Shortened lifespan is observed even under caloric restriction and high-fat diet feeding,^133^ mimicking susceptibility to external stressors similar to frailty.^4^^,^^134^ These phenotypes are almost reversed by inhibition of the FoxO pathway. The sarcopenic phenotypes are replicated in females, but the osteoporotic phenotypes are not.^133^

The genes knocked out in these models, together with the genes identified based on the GWASs in humans mentioned above, are associated with the hallmarks of aging, namely the nucleus, mitochondria, and nutrient anabolism, along with osteokines and myokines which exert anabolic or catabolic effects on bone and muscle, suggesting the potential significance of these factors in the development of osteosarcopenia (Figure 1).^6^^,^^9^ Some models suggest sex differences in the underlying mechanisms. Some die soon after developing osteosarcopenia, and others live with osteosarcopenia for months but eventually have a shortened lifespan. Some models show suppressed osteogenesis, others show enhanced osteolysis, and still others show both. Many of them exhibit lowered grip strength, but some exhibit also reduced exercise endurance, implying that this could be another key feature of (osteo-)sarcopenia, as previously proposed.^133^ Nonetheless, the common pathway mediating bone–muscle interactions remains to be identified. To our knowledge, no mouse models with osteoblast-, osteoclast-, or osteocyte-specific KOs (or over-expression) of genes potentially important for this process have been reported to exhibit osteosarcopenia, which would help to distinguish whether bone loss causes muscle loss or vice versa.

## Clinical care pathways and management

Managing osteosarcopenia requires a multimodal approach that targets both bone and muscle, often within geriatric or orthopedic care pathways (Figure 1). Major international guidelines consistently recommend comprehensive lifestyle interventions as the foundation of treatment, with pharmacotherapy added as needed for osteoporosis and sarcopenia.^135–137^

### Exercise

Exercise, particularly resistance exercise, is highly effective in managing and preventing sarcopenia and osteoporosis.^138^^,^^139^ Resistance training, especially when combined with optimal nutrition,^140^ is currently recommended as one of the most effective strategies to prevent (or reverse) sarcopenia and improve (or maintain) BMD.^141^^,^^142^ Strong evidence supports the benefits of balance and resistance exercises in reducing fall risk,^143^ reinforcing the idea: “stop the fall and you will prevent the fracture.” These benefits make resistance exercise particularly important when considering osteosarcopenia.^139^ Specifically, supervised high-intensity resistance exercises, tailored to individual ability and safety, have been shown to significantly enhance muscle strength and physical performance, and can modestly increase BMD.^144^ For example, the 18-mo FrOST trial demonstrated that a high-intensity resistance training program in older men with osteosarcopenia led to notable improvements in SMI and lower-limb strength, with trends toward maintained spine and hip BMD, compared to losses in the control group.^145^ The LIFTMOR and LIFTMOR-M trials clearly demonstrate that high-intensity resistance training improves not only lean mass and functional performance but also BMD in older adults, when they are appropriately supervised.^146^^,^^147^ A recent 2025 meta-analysis of exercise interventions in patients with osteosarcopenic adiposity (OS with obesity) found that exercise notably improved LS BMD (mean difference ~0.02 g/cm^2^ gain), increased grip strength, and reduced body fat, though it did not significantly affect gait speed or muscle mass in the short term.^148^ Besides resistance training, comprehensive exercise programs for osteosarcopenia should incorporate balance, gait, and functional training to prevent falls and support daily activities. Weight-bearing and agility exercises may further enhance bone health and coordination, while gentler options like chair rises, resistance bands, or aquatic therapy are suitable for frailer individuals. The emphasis should be on consistency, progression, and supervision. Key outcomes to monitor include gains in muscle strength, improved gait speed or SPPB scores, and maintenance or enhancement of BMD on follow-up DXA.

### Nutrition

Nutrition plays a pivotal role in both muscle and bone health, and among them, amino acids serve not only as the raw material for proteins but also activate signaling involved in protein anabolism.^11^ International working groups recommend higher protein intakes for older adults with sarcopenia: generally up to 1.2-1.8 g/kg/d if sarcopenia or weight loss is present.^149^ In osteosarcopenia patients, ensuring sufficient protein and caloric intake is fundamental; many are malnourished or have anorexia of aging.^150^ Trials have shown that added protein can augment strength gains and preserve lean mass during exercise programs.^151^ A recent clinical trial reported that high-intensity dynamic resistance training combined with whey protein supplementation significantly improved muscle mass, strength, and functional performance in older men with osteosarcopenia. The intervention also led to meaningful gains in BMD.^151^

Vitamin D and calcium supplementation may be an important component of a comprehensive intervention in individuals with osteosarcopenia who have vitamin D deficiency or inadequate dietary calcium intake. Vitamin D deficiency is common in older adults and is associated with muscle weakness and bone loss. Supplementing vitamin D to reach serum 25OHD levels above 75 nmol/L (30 ng/mL) is recommended, along with calcium intake of approximately 1000-1200 mg/d (from diet and supplements) to support bone mineralization.^150^ In the FROST study mentioned, both vitamin D (800 IU/d) and high protein intake (1.5-1.6 g/kg body mass/d) were provided to participants, resulting in positive outcomes for muscle and bone health.^152^

Other nutrients of interest include omega-3 fatty acids (which may exert anti-inflammatory effects on muscle), creatine (commonly studied as an adjunct to exercise to enhance muscle performance), and β-hydroxy-β-methylbutyrate (HMB, which may reduce muscle protein breakdown and enhance regeneration), which has been shown to improve muscle mass, strength, and function in older adults.^153^ However, the DO-HEALTH trial failed to show a reduction in fracture incidence with omega-3 or vitamin D supplementation.^154^

Moreover, recent evidence demonstrates the importance of other aspects of diet. For example, a diet rich in vegetables and grain will provide a range of other nutrients (eg, vitamin K, nitrate, and magnesium) that likely play a role in supporting bone metabolism and muscle strength.^140^ It is also worth noting that obtaining the recommended amount of calcium and vitamin D from wholefoods can be challenging in older adults with reduced appetite. In some cases, an overemphasis to meet recommendations for calcium intake from dairy foods may come at the expense of other food groups (eg, grain and vegetables), critical for other aspects of healthy aging. This is an important consideration where supplementation under the guidance of health care professionals (eg, clinicians and dietitians) may be warranted. It is important that nutritional interventions be personalized: for an obese osteosarcopenic patient, the focus might be on high-quality protein with calorie control; for a frail underweight patient, general caloric enrichment.

### Pharmacotherapy for osteoporosis

Currently, there is no pharmacological treatment proven to simultaneously preserve or enhance muscle mass, strength, and physical function. However, the bone component of osteosarcopenia should be managed according to established osteoporosis guidelines to reduce fracture risk. Antiresorptive agents, including bisphosphonates (eg, alendronate and zoledronic acid) and denosumab remain the first-line therapies for improving BMD and preventing fragility fractures in this population. Anabolic agents like teriparatide (PTH analog) or romosozumab (sclerostin inhibitor) can be used in severe osteoporosis or in those who fracture despite bisphosphonates.^155^ These drugs stimulate new bone formation and have been shown to improve BMD rapidly; interestingly, preliminary research suggests that they may also have positive musculoskeletal crosstalk effects (eg, PTH may influence muscle, and romosozumab affects bone and perhaps muscle mass via the Wnt pathway).^156^ If patients with osteosarcopenia are receiving catabolic medications for other diseases, it would be important to assess the balance between expected benefits and potential effects on bone and muscle on a regular basis.

### Integrated multidisciplinary care models

Because osteosarcopenia combines 2 related conditions, integrated multidimensional care models are recommended. The current consensus for managing chronic diseases in older adults is to adopt person-centered integrated care, bringing together multiple disciplines. The care pathway generally includes identifying at-risk patients (eg, an older individual who has experienced a minor fall or has known osteopenia), conducting a combined assessment as described earlier, initiating lifestyle measures (such as exercise classes and dietary counseling), and making appropriate referrals. The primary aim of integrated care is to preserve function and independence while preventing fractures.^157^ Educating healthcare professionals is very important; primary care physicians should be alert to muscle weakness in their osteoporotic patients and vice versa.

## Emerging strategies and future directions

On the diagnostic front, technological innovations are expected to enhance early and precise detection, but AI software and deep learning models are making a rapid progress far beyond. One of the frontiers in this field is AI-based opportunistic imaging analysis. For instance, AI software can calculate muscle area and myosteatosis on abdominal CT scans or estimate ALM from hip X-rays, and alert clinicians to sarcopenia, prompting further evaluation.^53^^,^^158^^,^^159^ Similarly, vertebral BMD can be derived from chest CTs.^160^ Moreover, such opportunistic imaging analysis is aiming even to estimate disease risks. For example, it was proposed to use whole body DXA data to predict the risk of falls,^161^ and to use CT or PET/CT data to predict the risk of mortality.^67^ Integration of imaging data, laboratory data, and medical history is expected to further help clinicians diagnose with osteosarcopenia.

In the laboratory domain, researchers are investigating circulating biomarkers that could serve as early warning signals of osteosarcopenia or as markers of response to therapy.^162^ Omics analyses are expected to help identify novel biomarkers even in the field of osteosarcopenia. Indeed, a novel metabolite, orotate, and a circulating protein-based risk score were recently reported to be associated with osteoporotic fracture.^163^^,^^164^ The combination of the genomics and metabolomics data successfully proved that orotate is causal for fracture and suggested that the metabolite could be a good drug target.^163^ By contrast, the protein-based risk score was associated with fracture independently of the genetic determinants of the circulating protein levels.^164^ In this case, proteomics data were enough to predict the risk of fracture, and genomics data had no additional value to the score. Thus, it should be flexibly optimized which omics data to use on a case-by-case basis.

Emerging therapeutic strategies aim to target the muscle-bone unit collectively, with myostatin, a myokine as a muscle growth inhibitor. Blocking myostatin with monoclonal antibodies like bimagrumab has shown increases in muscle mass in trials, without any functional improvement.^165^ If an effective myostatin or ActRII inhibitor is developed, it could be a game-changer for sarcopenia treatment and might also positively influence bone, just as an osteosarcopenia mouse model was successfully treated with soluble ActRIIB antibody to block the signaling.^109^ On the bone side, new osteoanabolic drugs (like romosozumab to neutralize sclerostin) not only improve bone density but also appear to alter muscle signaling pathways; future research may clarify if these agents confer dual benefits. There is also interest in neuromuscular electrical stimulation and vibration therapy which can stimulate muscle and bone mechanotransduction. It would be further intriguing if the etiology of osteosarcopenia and the efficacy of therapies could be predicted based on genetic background and other environmental factors, enabling individualized optimization of osteosarcopenia care.

## Data Availability

No new data were generated or analyzed in support of this research.
